# A compendium of nonredundant short polymerase III promoters for CRISPR applications

**DOI:** 10.1093/plphys/kiaf294

**Published:** 2025-07-17

**Authors:** Michihito Deguchi, Kayla M Sinclair, Annie Patel, McKenna Coile, María A Ortega, William P Bewg, Chung-Jui Tsai

**Affiliations:** Warnell School of Forestry and Natural Resources, Department of Genetics, and Department of Plant Biology, University of Georgia, Athens, GA 30602, USA; Warnell School of Forestry and Natural Resources, Department of Genetics, and Department of Plant Biology, University of Georgia, Athens, GA 30602, USA; Warnell School of Forestry and Natural Resources, Department of Genetics, and Department of Plant Biology, University of Georgia, Athens, GA 30602, USA; Warnell School of Forestry and Natural Resources, Department of Genetics, and Department of Plant Biology, University of Georgia, Athens, GA 30602, USA; Warnell School of Forestry and Natural Resources, Department of Genetics, and Department of Plant Biology, University of Georgia, Athens, GA 30602, USA; Warnell School of Forestry and Natural Resources, Department of Genetics, and Department of Plant Biology, University of Georgia, Athens, GA 30602, USA; Warnell School of Forestry and Natural Resources, Department of Genetics, and Department of Plant Biology, University of Georgia, Athens, GA 30602, USA

## Abstract

Minimal native and synthetic Polymerase III promoters enable efficient and customizable CRISPR multiplexing in plants, expanding genome engineering capabilities

Dear Editor,

Multiplex genome editing via CRISPR is a powerful tool for simultaneous knockout, activation, and/or repression of distinct genes. However, current toolkits for multiplex editing lack diversity. Polymerase III (Pol III) promoters are widely used to express guide RNAs (gRNAs). Repeated sequences, including promoters, in multiple expression cassettes complicate construct assembly and have long been a concern for genetic stability and unwanted silencing (Assaad et al. 1993; Peremarti et al. 2010). Expressing gRNAs as a polycistronic array eliminates the need for multiple promoters, but these strategies still involve repeated use of tRNAs, ribozymes, or other RNA-cleaving systems (Xie et al. 2015; Čermák et al. 2017), raising additional concerns about variable gRNA processing. Furthermore, using unnecessarily long promoters may increase the genetic load and introduce uncertainties that impact CRISPR efficiency. Here, we present a diverse collection of short Pol III promoters to support increasingly sophisticated genome editing applications in dicots.

Published plant U6 and U3 promoters range from 79 bp (Nekrasov et al. 2013) to over 700 bp (Ma et al. 2015; Dai et al. 2021) (Supplementary Table S1), with longer promoters being several fold larger than the transcripts they control. Because the upstream sequence element (USE) and TATA box necessary for Pol III transcription are close to the transcription start site (Waibel and Filipowicz 1990), we reasoned that minimal Pol III promoters would be effective. We assessed Pol III promoter activity for CRISPR-Cas9 editing of *mEGFP* (monomeric enhanced green fluorescent protein) in stably retransformed *Nicotiana benthamiana* and poplar (*Populus tremula* × *alba* INRA 717-1B4) reporter lines (Supplementary Text S1). We first tested a deletion series of the experimentally validated *Medicago truncatula MtU6.6* promoter (352 bp; Zhou et al. 2015). The 70 bp *MtU6.6* promoter was as effective as the 352 bp promoter, based on the loss of mEGFP signal in both root tips and leaf trichomes of *N. benthamiana* (Fig. 1A). Amplicon sequencing confirmed that all 4 promoter lengths had similar efficiencies in *N. benthamiana* and poplar, whereas the promoterless control was ineffective (Fig. 1B; Supplementary Table S2).

**Figure 1.**
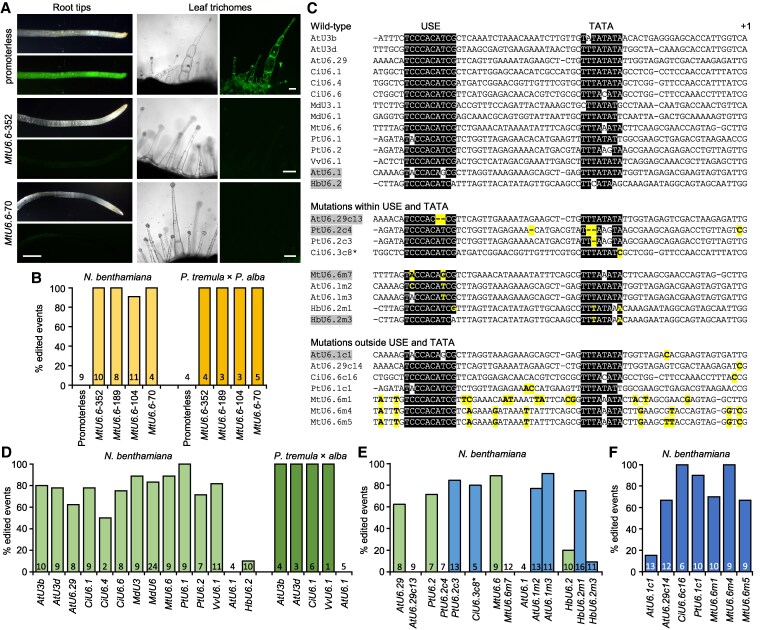
Pol III promoter characterization. **A)** Images of *N. benthamiana* root tips or leaf trichomes under brightfield or UV. Scale bars: 1 mm (root tips), 100 µm (trichomes). **B)** Editing efficiencies of the *MtU6.6* promoter deletion series. **C)** Alignments of 70 bp promoters. Conserved USE and TATA box sequences are shaded in black, mutations in yellow, and nonfunctional promoters in gray. **D)** Editing efficiencies of 70 bp promoters from diverse origins. **E)** Editing efficiencies of 70 bp promoters with mutations in the USE or TATA (blue bars). Wild-type promoter data (from **D)** light green bars) are included as reference. **F)** Editing efficiencies of 70 bp promoters with mutations outside USE and TATA. Transgenic event numbers are indicated at the bottom of the bars. *CiU6.3 wild-type promoter was not tested. At, *Arabidopsis thaliana*; Ci, *Cichorium intybus*; Hb, *Hevea brasiliensis*; Md, *Malus domestica*; Mt, *Medicago truncatula*; Pt, *Populus trichocarpa*; Vv, *Vitis vinifera*.

We next investigated whether the 70 bp length is universally effective for dicot Pol III promoters. We oligo-synthesized 14 short U6/U3 promoters of diverse origin (Fig. 1C; Supplementary Table S3) for construct assembly. They were selected with either demonstrated or inconsistent activities (Li et al. 2013, 2021; Fan et al. 2015; Ma et al. 2015) or identified from the black cottonwood (*Populus trichocarpa*) genome with unknown functionality (Supplementary Table S1). All but 2 (*Arabidopsis thaliana AtU6.1* and *Hevea brasiliensis HbU6.2*) showed efficient editing in *N. benthamiana* (Fig. 1D). The *AtU6.1* promoter also failed in poplar, but the other 4 short promoters tested were functional. These results suggest that the 70 bp length is sufficient for gRNA expression in dicots, with some exceptions.

One of the exceptions, *AtU6.1*, was also reported as ineffective in different *Populus* species (Fan et al. 2015; Li et al. 2021), suggesting a genetic basis for the defect. Close examination revealed sequence variations within the USE and/or TATA box of *AtU6.1* and *HbU6.2* (Fig. 1C). To demonstrate causality, we utilized erroneous promoter clones from multiplex Gibson assembly of pooled oligos. We found that 2 nt deletions within the USE (*AtU6.29c13*) or TATA box (*PtU6.2c4*) abolished editing, whereas a single-base deletion in the TATA box (*PtU6.2c3*) was functional (Fig. 1E). Mutations outside the conserved elements did not impact activity (Fig. 1F). Next, we generated reciprocal mutations between the functional *MtU6.6* and the defective *AtU6.1* targeting the 2 variable USE positions. Mutation of the *MtU6.6* USE (*MtU6.6m7*_C2A/T8G_) to resemble *AtU6.1* resulted in a complete loss of activity, whereas the reciprocal mutation of *AtU6.1* (*AtU6.1m2*_A2C/G8T_) restored editing (Fig. 1E). Correction at the eighth position alone (*AtU6.1m3*_G8T_) to resemble the USE of *PtU6.1* was sufficient to restore activity, indicating the T8G mutation as causal to the *AtU6.1* promoter defect. For *HbU6.2*, mutations to restore USE (A10G) and TATA box (C3T/G8A) conservation, but not the TATA box alone, rescued promoter activity (Fig. 1E). This suggests that the A10G mutation of USE is causal to *HbU6.2* inactivity. It is worth noting that some variations in the AT-rich TATA box appeared tolerated, indicative of a less stringent requirement.

The observation that sequences outside the USE and TATA elements are highly variable suggests that nonconserved regions can be exploited to generate synthetic Pol III promoters. We designed 2 *MtU6.6* promoter variants with 9 to 13 dispersed substitutions outside the USE and TATA elements (*MtU6.6m1* and *MtU6.6m4*). A third variant, *MtU6.6m5*, was a cloning artifact with an additional substitution compared to *MtU6.6m4* (Fig. 1C). All 3 variants retained promoter activity (Fig. 1F), supporting the idea that sequences outside USE and TATA can be altered without affecting function.

Finally, we DNA-synthesized a 4-plex design using nonredundant promoters of varying lengths for one-step binary vector cloning to target trichome-regulating *MYB* paralogs in poplar (Fig. 2A and B; Supplementary Text S1) (Bewg et al. 2022). Mutations at all target sites were evidenced by the glabrous phenotype and confirmed by amplicon sequencing in transgenic poplar, demonstrating that short promoters are effective in multiplex editing (Fig. 2C and D; Supplementary Table S2). This simple and cost-effective approach of synthesizing multi-gRNA cassettes with short, nonredundant U6/U3 promoters for multiplex construct assembly has also been reported elsewhere (Ortega et al. 2023; Nagy et al. 2025).

**Figure 2.**
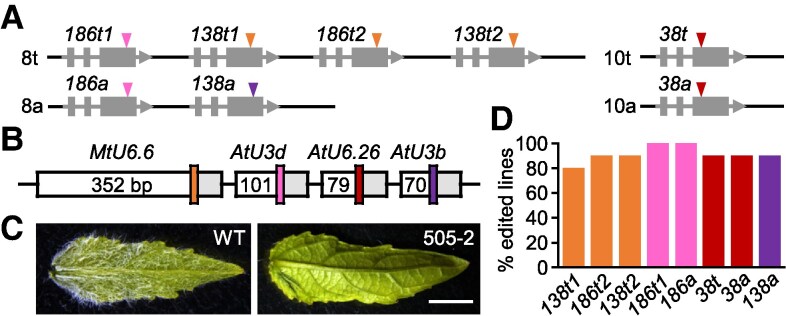
Multiplex editing using nonredundant promoters. **A)** Schematic representation of the 8 *MYB186*, *MYB138*, and *MYB38* alleles on chromosomes 8 and 10 of the *P. tremula* (t) and *P. alba* (a) subgenomes. Colored arrowheads indicate the target sites of 4 gRNAs. **B)** Diagram of the construct design showing 4 color-coded gRNAs driven by distinct promoters of varying lengths (bp). **C)** Representative leaf images of wild-type (WT) and an edited plant. Scale bar: 0.5 cm. **D)** Editing efficiencies at the 8 target alleles across *n* = 10 transgenic lines, with alleles color-coded by gRNA as in panels **(A** and **B)**.

In summary, this study presents several advances and lessons regarding Pol III promoters. We demonstrate the effectiveness of minimal U6 and U3 promoters from various dicot species. The USE consensus can be refined as TMCCACATCG (M = A or C), whereas the TATA consensus is less strict. Not all wild-type Pol III promoters are functional due to USE variation, as shown for *AtU6.1* and *HbU6.2*. The updated USE and TATA consensus, along with the mutagenesis data presented, can guide the selection of Pol III promoters with broad functionality. We also exemplified the design of new-to-nature promoters to increase diversity. Given the nonconserved sequence space and combinatorial nucleotide possibilities, the potential for synthetic Pol III promoters is immense. Although this work focused on dicots, similar approaches are applicable to monocot Pol III promoters, which harbor additional monocot-specific cis elements upstream of USE (Hao et al. 2020; Nagy et al. 2025). The compendium of 23 experimentally verified dicot U6/U3 promoters should enhance multiplex genome editing capability for CRISPR applications.

## Supplementary Material

kiaf294_Supplementary_Data

## Data Availability

The data underlying this article are available in the article and in its online supplementary material.

## Supplementary data

The following materials are available in the online version of this article.

**Supplementary Table S1.** Published U6 and U3 promoters from dicot species.

**Supplementary Table S2.** Amplicon sequencing results of the transgenic lines.

**Supplementary Table S3.** Oligonucleotide and DNA sequences used in this study.

## References

[kiaf294-B1] Assaad FF, Tucker KL, Signer ER. Epigenetic repeat-induced gene silencing (RIGS) inArabidopsis. Plant Mol Biol. 1993:22(6):1067–1085. 10.1007/BF00028978 8400126

[kiaf294-B2] Bewg WP, Harding SA, Engle NL, Vaidya BN, Zhou R, Reeves J, Horn TW, Joshee N, Jenkins JW, Shu S, et al Multiplex knockout of trichome-regulating MYB duplicates in hybrid poplar using a single gRNA. Plant Physiol. 2022:189(2):516–526. 10.1093/plphys/kiac128 35298644 PMC9157173

[kiaf294-B3] Čermák T, Curtin SJ, Gil-Humanes J, Čegan R, Kono TJY, Konečná E, Belanto JJ, Starker CG, Mathre JW, Greenstein RL, et al A multipurpose toolkit to enable advanced genome engineering in plants. Plant Cell. 2017:29(6):1196–1217. 10.1105/tpc.16.00922 28522548 PMC5502448

[kiaf294-B4] Dai X, Yang X, Wang C, Fan Y, Xin S, Hua Y, Wang K, Huang H. CRISPR/Cas9-mediated genome editing in *Hevea brasiliensis*. Ind Crops Prod. 2021:164:113418. 10.1016/j.indcrop.2021.113418

[kiaf294-B5] Fan D, Liu T, Li C, Jiao B, Li S, Hou Y, Luo K. Efficient CRISPR/Cas9-mediated targeted mutagenesis in *Populus* in the first generation. Sci Rep. 2015:5(1):12217. 10.1038/srep12217 26193631 PMC4507398

[kiaf294-B6] Hao Y, Zong W, Zeng D, Han J, Chen S, Tang J, Zhao Z, Li X, Ma K, Xie X, et al Shortened snRNA promoters for efficient CRISPR/Cas-based multiplex genome editing in monocot plants. Sci China Life Sci. 2020:63(6):933–935. 10.1007/s11427-019-1612-6 31942685

[kiaf294-B7] Li G, Sretenovic S, Eisenstein E, Coleman G, Qi Y. Highly efficient C-to-T and A-to-G base editing in a Populus hybrid. Plant Biotechnol J. 2021:19(6):1086–1088. 10.1111/pbi.13581 33742755 PMC8196628

[kiaf294-B8] Li J-F, Norville JE, Aach J, McCormack M, Zhang D, Bush J, Church GM, Sheen J. Multiplex and homologous recombination–mediated genome editing in *Arabidopsis* and *Nicotiana benthamiana* using guide RNA and Cas9. Nat Biotechnol. 2013:31(8):688–691. 10.1038/nbt.2654 23929339 PMC4078740

[kiaf294-B9] Ma X, Zhang Q, Zhu Q, Liu W, Chen Y, Qiu R, Wang B, Yang Z, Li H, Lin Y, et al A robust CRISPR/Cas9 system for convenient, high-efficiency multiplex genome editing in monocot and dicot plants. Mol Plant. 2015:8(8):1274–1284. 10.1016/j.molp.2015.04.007 25917172

[kiaf294-B10] Nagy ED, Davis IW, Song S, No V, Wu C, Kanizay L, Turner-Hissong S, Li H, Ye X, Berry JC, et al Computationally derived RNA polymerase III promoters enable maize genome editing. Front Plant Sci. 2025:16:1540425. 10.3389/fpls.2025.1540425 40177017 PMC11961915

[kiaf294-B11] Nekrasov V, Staskawicz B, Weigel D, Jones JDG, Kamoun S. Targeted mutagenesis in the model plant Nicotiana benthamiana using Cas9 RNA-guided endonuclease. Nat Biotechnol. 2013:31(8):691–693. 10.1038/nbt.2655 23929340

[kiaf294-B12] Ortega MA, Zhou R, Chen MSS, Bewg WP, Simon B, Tsai C-J. In vitro floral development in poplar: insights into seed trichome regulation and trimonoecy. New Phytol. 2023:237(4):1078–1081. 10.1111/nph.18624 36385612 PMC10107547

[kiaf294-B13] Peremarti A, Twyman RM, Gómez-Galera S, Naqvi S, Farré G, Sabalza M, Miralpeix B, Dashevskaya S, Yuan D, Ramessar K, et al Promoter diversity in multigene transformation. Plant Mol Biol. 2010:73(4–5):363–378. 10.1007/s11103-010-9628-1 20354894

[kiaf294-B14] Waibel F, Filipowicz W. U6 snRNA genes of Arabidopsis are transcribed by RNA polymerase III but contain the same two upstream promoter elements as RNA polymerase ll-transcribed U-snRNA genes. Nucleic Acids Res. 1990:18(12):3451–3458. 10.1093/nar/18.12.3451 2362802 PMC330996

[kiaf294-B15] Xie K, Minkenberg B, Yang Y. Boosting CRISPR/Cas9 multiplex editing capability with the endogenous tRNA-processing system. Proc Natl Acad Sci U S A. 2015:112(11):3570–3575. 10.1073/pnas.1420294112 25733849 PMC4371917

[kiaf294-B16] Zhou X, Jacobs TB, Xue L-J, Harding SA, Tsai C-J. Exploiting SNPs for biallelic CRISPR mutations in the outcrossing woody perennial *Populus* reveals 4-coumarate:CoA ligase specificity and redundancy. New Phytol. 2015:208(2):298–301. 10.1111/nph.13470 25970829

