# Supplementary material for: A compendium of nonredundant short polymerase III promoters for CRISPR applications
Source: Plant Physiol. 2025 Jul 17;198(3):kiaf294. doi: 10.1093/plphys/kiaf294 (PMC12268498; doi:10.1093/plphys/kiaf294)
Supplement: kiaf294_Supplementary_Data [file kiaf294_supplementary_data.zip › SupplementaryMaterial.pdf]

**Supplementary Table S1. Published U6 and U3 promoters from dicot species**

| Pol III promoter | Source species | Length (bp) | Reference |
|---|---|---|---|
| AtU3b | *Arabidopsis thaliana* | 326 | Ma et al. 2015 |
| AtU3b | *Arabidopsis thaliana* | 153 | Ren et al. 2021 |
| AtU3b | *Arabidopsis thaliana* | 70 | this study |
| AtU3d | *Arabidopsis thaliana* | 101 | Ma et al. 2015 |
| AtU3d | *Arabidopsis thaliana* | 70 | this study |
| AtU6-1 | *Arabidopsis thaliana* | 304 | Li et al. 2013; Fan et al. 2015 |
| AtU6-1 | *Arabidopsis thaliana* | 249 | Li et al. 2021 |
| AtU6.1 | *Arabidopsis thaliana* | 70 | this study |
| AtU6.1c1 | *Arabidopsis thaliana* | 70 | this study |
| AtU6.1m2 | *Arabidopsis thaliana* | 70 | this study |
| AtU6.1m3 | *Arabidopsis thaliana* | 70 | this study |
| AtU6-26 | *Arabidopsis thaliana* | 448 | Feng et al. 2013 |
| AtU6-26 | *Arabidopsis thaliana* | 424 | Xing et al. 2014 |
| AtU6-26 | *Arabidopsis thaliana* | 387 | Fauser et al. 2014 |
| AtU6-26 | *Arabidopsis thaliana* | 299 | Mao et al. 2013 |
| AtU6-26 | *Arabidopsis thaliana* | 205 | Castel et al. 2019 |
| AtU6.26 | *Arabidopsis thaliana* | 79 | Nekrasov et al. 2013 |
| AtU6-29 | *Arabidopsis thaliana* | 319 | Ma et al. 2015 |
| AtU6-29 | *Arabidopsis thaliana* | 285 | Xing et al. 2014 |
| AtU6-29 | *Arabidopsis thaliana* | 70 | this study |
| AtU6.29c14 | *Arabidopsis thaliana* | 70 | this study |
| AtU6.29c13 | *Arabidopsis thaliana* | 70 | this study |
| CiU6-1 | *Cichorium intybus* | 206 | Bernard et al. 2019 |
| CiU6.1 | *Cichorium intybus* | 70 | this study |
| CiU6.3 | *Cichorium intybus* | 299 | Bernard et al. 2019 |
| CiU6.3c8 | *Cichorium intybus* | 70 | this study |
| CiU6.4 | *Cichorium intybus* | 302 | Bernard et al. 2019 |
| CiU6.4 | *Cichorium intybus* | 70 | this study |
| CiU6.6 | *Cichorium intybus* | 302 | Bernard et al. 2019 |
| CiU6.6 | *Cichorium intybus* | 70 | this study |
| CiU6.6c16 | *Cichorium intybus* | 70 | this study |
| CsU3-1 | *Camelina sativa* | 335 | Morineau et al., 2016 |
| CsU6-1 | *Camelina sativa* | 332 | Morineau et al., 2016 |
| GhU6.3 | *Gossypium hirsutum* | 300 | Long et al., 2018 |

| **Pol III promoter** | **Source species** | **Length (bp)** | **Reference** |
|---|---|---|---|
| GmU6.10 | *Glycine max* | 307 | Sun et al., 2015 |
| HbU6.1 | *Hevea brasiliensis* | 694 | Dai et al., 2021 |
| HbU6.2 | *Hevea brasiliensis* | 345 | Dai et al., 2021 |
| HbU6.2 | *Hevea brasiliensis* | 70 | this study |
| HbU6.2m1 | *Hevea brasiliensis* | 70 | this study |
| HbU6.2m3 | *Hevea brasiliensis* | 70 | this study |
| HbU6-3 | *Hevea brasiliensis* | 546 | Dai et al., 2021 |
| HbU6-4 | *Hevea brasiliensis* | 462 | Dai et al., 2021 |
| HbU6-5 | *Hevea brasiliensis* | 754 | Dai et al., 2021 |
| MdU3.1 | *Malus x domestica* | 327 | Charrier et al., 2019 |
| MdU3.1 | *Malus x domestica* | 70 | this study |
| MdU6.1 | *Malus x domestica* | 324 | Charrier et al., 2019 |
| MdU6.1 | *Malus x domestica* | 70 | this study |
| MtU6.6 | *Medicago truncatula* | 352 | Jacob et al. 2015 |
| MtU6.6 | *Medicago truncatula* | 189 | this study |
| MtU6.6 | *Medicago truncatula* | 104 | this study |
| MtU6.6 | *Medicago truncatula* | 70 | this study |
| MtU6.6m1 | *Medicago truncatula* | 70 | this study |
| MtU6.6m4 | *Medicago truncatula* | 70 | this study |
| MtU6.6m5 | *Medicago truncatula* | 70 | this study |
| MtU6.6m7 | *Medicago truncatula* | 70 | this study |
| NtU6.1 | *Nicotiana tabacum* | 299 | Luo et al., 2024 |
| NtU6.2 | *Nicotiana tabacum* | 276 | Luo et al., 2024 |
| NtU6.3 | *Nicotiana tabacum* | 299 | Luo et al., 2024 |
| PtU6.1 | *Populus trichocarpa*, upstream of Potri.005G055401 | 70 | this study |
| PtU6.1c1 | *Populus trichocarpa* | 70 | this study |
| PtU6.2 | *Populus trichocarpa*, upstream of Potri.005G055401 | 70 | this study |
| PtU6.2c3 | *Populus trichocarpa* | 70 | this study |
| PtU6.2c4 | *Populus trichocarpa* | 70 | this study |
| VvU3.1 | *Vitis vinifera* | 393 | Ren et al. 2021 |
| VvU3.2 | *Vitis vinifera* | 511 | Ren et al. 2021 |
| VvU6.1 | *Vitis vinifera* | 425 | Ren et al. 2021 |
| VvU6.1 | *Vitis vinifera* | 70 | this study |
| VvU6.2 | *Vitis vinifera* | 591 | Ren et al. 2021 |

**References**


**Bernard G, Gagneul D, Alves Dos Santos H, Etienne A, Hilbert J-L, Rambaud C** (2019) Efficient genome editing using CRISPR/Cas9 technology in chicory. International Journal of Molecular Sciences **20:** 1155

**Castel B, Tomlinson L, Locci F, Yang Y, Jones JDG** (2019) Optimization of T-DNA architecture for Cas9-mediated mutagenesis in *Arabidopsis*. PLOS ONE **14:** e0204778

**Charrier A, Vergne E, Dousset N, Richer A, Petiteau A, Chevreau E** (2019) Efficient targeted mutagenesis in apple and first time edition of pear using the CRISPR-Cas9 system. Frontiers in Plant Science **10:** 40

**Dai X, Yang X, Wang C, Fan Y, Xin S, Hua Y, Wang K, Huang H** (2021) CRISPR/Cas9-mediated genome editing in Hevea brasiliensis. Industrial Crops and Products **164:** 113418

**Fan D, Liu T, Li C, Jiao B, Li S, Hou Y, Luo K** (2015) Efficient CRISPR/Cas9-mediated targeted mutagenesis in *Populus* in the first generation. Scientific Reports **5:** 12217

**Fauser F, Schiml S, Puchta H** (2014) Both CRISPR/Cas-based nucleases and nickases can be used efficiently for genome engineering in *Arabidopsis thaliana*. Plant J **79:** 348-359

**Feng ZY, Zhang BT, Ding WN, Liu XD, Yang DL, Wei PL, Cao FQ, Zhu SH, Zhang F, Mao YF, Zhu JK** (2013) Efficient genome editing in plants using a CRISPR/Cas system. Cell Research **23:** 1229-1232

**Jacobs TB, LaFayette PR, Schmitz RJ, Parrott WA** (2015) Targeted genome modifications in soybean with CRISPR/Cas9. BMC Biotechnology **15:** 16

**Li J-F, Norville JE, Aach J, McCormack M, Zhang D, Bush J, Church GM, Sheen J** (2013) Multiplex and homologous recombination–mediated genome editing in *Arabidopsis* and *Nicotiana benthamiana* using guide RNA and Cas9. Nature Biotechnology **31:** 688-691

**Li G, Sretenovic S, Eisenstein E, Coleman G, Qi Y** (2021) Highly efficient C-to-T and A-to-G base editing in a Populus hybrid. Plant Biotechnology Journal **19:** 1086-1088

**Long L, Guo DD, Gao W, Yang WW, Hou LP, Ma XN, Miao YC, Botella JR, Song CP** (2018) Optimization of CRISPR/Cas9 genome editing in cotton by improved sgRNA expression. Plant Methods **14:** 85

**Luo P, Li S, Li L, Li Y, Qiao Y, Wu X, Wu M, Song Z, Yang Y, Feng J, Tang X** (2024) Validation of endogenous U6 promoters for expanding the CRISPR toolbox in *Nicotiana tabacum*. *In Vitro* Cellular & Developmental Biology - Plant **60:** 378-383

**Ma X, Zhang Q, Zhu Q, Liu W, Chen Y, Qiu R, Wang B, Yang Z, Li H, Lin Y, Xie Y, Shen R, Chen S, Wang Z, Chen Y, Guo J, Chen L, Zhao X, Dong Z, Liu Y-G** (2015) A robust CRISPR/Cas9 system for convenient, high-efficiency multiplex genome editing in monocot and dicot plants. Molecular Plant **8:** 1274-1284

**Mao Y, Zhang H, Xu N, Zhang B, Gou F, Zhu J-K** (2013) Application of the CRISPR–Cas system for efficient genome engineering in plants. Molecular Plant **6:** 2008-2011

**Morineau C, Bellec Y, Tellier F, Gissot L, Kelemen Z, Nogué F, Faure J-D** (2017) Selective gene dosage by CRISPR-Cas9 genome editing in hexaploid *Camelina sativa*. Plant Biotechnology Journal **15:** 729-739

**Nekrasov V, Staskawicz B, Weigel D, Jones JDG, Kamoun S** (2013) Targeted mutagenesis in the model plant Nicotiana benthamiana using Cas9 RNA-guided endonuclease. Nature Biotechnology **31:** 691-693

**Ren C, Liu X, Zhang Z, Wang Y, Duan W, Li S, Liang Z** (2016) CRISPR/Cas9-mediated efficient targeted mutagenesis in Chardonnay (*Vitis vinifera* L.). Scientific Reports **6:** 32289

**Sun X, Hu Z, Chen R, Jiang Q, Song G, Zhang H, Xi Y** (2015) Targeted mutagenesis in soybean using the CRISPR-Cas9 system. Scientific Reports **5:** 10342

**Xing HL, Dong L, Wang ZP, Zhang HY, Han CY, Liu B, Wang XC, Chen QJ** (2014) A CRISPR/Cas9 toolkit for multiplex genome editing in plants. BMC Plant Biol **14:** 327

**Supplementary Table S3.** Oligonucleotide and DNA sequences used in this study

| Name | Sequence |
|---|---|
| ***Primers for cloning*** | |
| Rps5A.SpepORE.F | CCGAGTCGGTGCTTTTTTTACTAGTGGAGCTCAACTTTTGATTCGCT |
| Rps5a.NhepORE.R | TGGAGTACTTCTTGTCCATGCTAGCTGCTGTGGTGAGAGAAACAG |
| eGFP.g264U6 | CAAGCGAACCAGTAGGCTTGCATGCCCGAAGGCTACGTCC |
| eGFP.g264SF | GCTATTTCTAGCTCTAAAACGGACGTAGCCTTCGGGCATG |
| RPS5A29R | GCACTGCAAATAGCGAATCAA |
| StubiPKpnIeGFP | GATATTAATCTCTTCGATGAAGGTACCGCATGCCCGAAGGCTACGTCC |
| MtU6.189F | GATATTAATCTCTTCGATGAACCTAGGACAAGGTGCACTGCTACAA |
| MtU6.104F | GATATTAATCTCTTCGATGAACCTAGGCGAGAAGGATTTGCATGTTACT |
| MtU6.70F | GATATTAATCTCTTCGATGAACCTAGGTTTTAGTCCCACATCGTCTGAAA |
| eGFP.g264U6rc | GGACGTAGCCTTCGGGCATGCAAGCCTACTGGTTCGCTTG |
| ***Primers for amplicon library preparation*** | |
| eGFP187tailF | CCTACACGACGCTCTTCCGATCTACCACCCTGACCTACGG |
| eGFP449tailR | GTTCAGACGTGTGCTCTTCCGATCTGTGGCTGTTGTAGTTGTACTC |
| Fuzzy37F.tailF4 | TTTCCCTACACGACGCTCTTCCGATCTCMAAAGCTGATTCTTTCGGCTC |
| Fuzzy37R.tailR4 | GAGTTCAGACGTGTGCTCTTCCGATCTAGCATTATTTGTGGCAAAAGCTAGT |
| Fuzzy46F.tailF4 | TTTCCCTACACGACGCTCTTCCGATCTTAGCTTTTGCCACAAATAATGCT |
| Fuzzy46R.tailR4 | GAGTTCAGACGTGTGCTCTTCCGATCTCCCTSARTCAAAYCCTACAA |
| ***Short promoter oligos*** | |
| MtU6-70GFP264 | CGATATTAATCTCTTCGATGAAGGCCTTTTTAGTCCCACATCGTCTGAAACATAAAATATT<br>TCAGCGTTTAAATACTTCAAGCGAACCAGTAGGCTTGCATGCCCGAAGGCTACGTCC |
| AtU6.1-70GFP264 | CGATATTAATCTCTTCGATGAAGGCCTCAAAAGTACCACAGCGCTTAGGTAAAGAAAGCAG<br>CTGAGTTTATATATGGTTAGAGACGAAGTAGTGATTGCATGCCCGAAGGCTACGTCC |
| VvU6.1-70GFP264 | CGATATTAATCTCTTCGATGAAGGCCTACTCTTCCCACATCGACTGCTCATAGACGAAATT<br>GAGCTTTTATATATCAGGAGCAAACGCTTAGAGCTTGCATGCCCGAAGGCTACGTCC |
| CiU6.1-70GFP264 | CGATATTAATCTCTTCGATGAAGGCCTATGGCTTCCCACATCGCTCATTGGAGCAACATCG<br>CCATGCTTTATATAGCCTCGCCTCCAACCATTTATCGCATGCCCGAAGGCTACGTCC |
| AtU3d-70GFP264 | CGATATTAATCTCTTCGATGAAGGCCTTTTGCGTCCCACATCGGTAAGCGAGTGAAGAAAT<br>AACTGCTTTATATATGGCTACAAAGCACCATTGGTCACATGCCCGAAGGCTACGTCC |
| AtU3b-70GFP264 | CGATATTAATCTCTTCGATGAAGGCCTATTTCTCCCACATCGCTCAAATCTAAACAAATCT<br>TGTTGTATATATAACACTGAGGGAGCACCATTGGTCACATGCCCGAAGGCTACGTCC |
| PtU6-70GFP264 | CGATATTAATCTCTTCGATGAAGGCCTAGATATACCACATCGTCTGGTTAGAGAACACATG<br>AAGTTTTTATATTGGCGAAGCTGAGACGTAAGAACCGCATGCCCGAAGGCTACGTCC |
| HbU6-70GFP264 | CGATATTAATCTCTTCGATGAAGGCCTCTTAGTCCCACATCATTTAGTTACATATAGTTGC<br>AAGCCTTCATAAGCAAAGAATAGGCAGTAGCAATTGGCATGCCCGAAGGCTACGTCC |
| MdU6-70GFP264 | CGATATTAATCTCTTCGATGAAGGCCTGAGGTGTCCCACATCGAGCAAACGCAGTGGTATT<br>AATTGCTTTATATTCAATTAGACTGCAAAAAGTGTTGCATGCCCGAAGGCTACGTCC |

| | |
|---|---|
| MdU3-70GFP264 | CGATATTAATCTCTTCGATGAAGGCCTTTCTTGTCCCACATCGACCGTTTCCAGATTACTA<br>AAGCTGCTTATATGCCTAAACAATGACCAACTGTTCACATGCCCGAAGGCTACGTCC |
| AtU6.1C-70GFP264 | ATTAATCTCTTCGATGAAGGCCTCAAAAGTACCACAGCGCTTAGGTAAAGAAAGCAGCTGA<br>GTTTATATATGGTTAGACACGAAGTAGTGATTGCATGCCCGAAGGCTACGT |
| AtU6.26-70GFP264 | ATTAATCTCTTCGATGAAGGCCTAAAAGTCCCACATCGATCAGGTGATATATAGCAGCTTA<br>GTTTATATAATGATAGAGTCGACATAGCGATTGCATGCCCGAAGGCTACGT |
| AtU6.26b-70GFP264 | ATTAATCTCTTCGATGAAGGCCTAAAAGTCCCACATCGCTTAGATAAGAAAACGAAGCTGA<br>GTTTATATACAGCTAGAGTCGAAGTAGTGATTGCATGCCCGAAGGCTACGT |
| AtU6.29-70GFP264 | ATTAATCTCTTCGATGAAGGCCTAAAACATCCCACATCGTTCAGTTGAAAATAGAAGCTCT<br>GTTTATATATTGGTAGAGTCGACTAAGAGATTGCATGCCCGAAGGCTACGT |
| CiU6.3-70GFP264 | ATTAATCTCTTCGATGAAGGCCTATGGCTTCCCACATCGCTCTTTGAAACAACATCGTCAT<br>GCTTTATATAGACTCGCCTTCAACCATAGTTCGCATGCCCGAAGGCTACGT |
| CiU6.4-70GFP264 | ATTAATCTCTTCGATGAAGGCCTTGGCTCTCCCACATCGATGATCGGAACGGTTGTTTCGT<br>GCTTTATATAGCTCGGGTTCCAACCATTTATCGCATGCCCGAAGGCTACGT |
| CiU6.6-70GFP264 | ATTAATCTCTTCGATGAAGGCCTCTGGCTTCCCACATCGTTCATGGAGACAACACGTCTGC<br>GCTTTACATAGCCTGGCTTCCAAACCTTTATCGCATGCCCGAAGGCTACGT |
| MtU6m1-70GFP264 | ATTAATCTCTTCGATGAAGGCCTTTTATGTCCCACATCGTTCGAAACAATAAATTATTCAC<br>GGTTTAAATACTACTAGCGAACGAGTAGGCTTGCATGCCCGAAGGCTACGT |
| MtU6m1-70GFP264 | ATTAATCTCTTCGATGAAGGCCTTATTTGTCCCACATCGTCAGAAAGATAAATTATATCAG<br>CGTTTAAATACTTGAAGCGTACCAGTAGGGTTGCATGCCCGAAGGCTACGT |
| MtU6m2-70GFP264 | ATTAATCTCTTCGATGAAGGCCTTTATTGTCCCACATCGCTTGAAACTAAAAAATTTTCAG<br>GCTTTAAATACATCTAGCGAAGCAGTAGGCTTGCATGCCCGAAGGCTACGT |
| PtU6.2-70GFP264 | ATTAATCTCTTCGATGAAGGCCTAGATATCCCACATCGTCTGGTTAGAGAAAACATGACGT<br>ATTTAAGTAAGCGAAGCAGAGACCTGTTAGTTGCATGCCCGAAGGCTACGT |

***Synthetic DNA fragments for multiplex construct cloning***

| | |
|---|---|
| 4plexFuzzy6.1: MtU6.6tail-gRNA1-scaffold-linker1-AtU3d-gRNA2-scaffold-linker3 | TCAAGCGAACCAGTAGGCTTGAATTACCGGCACAAGTGAAAGTTTAAGAGCTATGCTGAAA<br>AGCATAGCAAGTTTAAATAAGGCTAGTCCGTTAaCAACggGAAAccGTGGCACCGAGTCGG<br>TGCTTTTTTTGCTACCTCAGCATAGTCTACAGCATAAGCTTATGATTTCTTTTTTCTTACG<br>AATTTTGCGTCCCACATCGGTAAGCGAGTGAAGAAATAACTGCTTTATATATGGCTACAAA<br>GCACCATTGGTCACATGATACAAGCCCGTTTGCGTTTCAGAGCTAGAAATAGCAAGTTGAA<br>ATAAGGCTAGTCCGTTATCACGCCGAAAGGCGGGCACCGAGTCGGTGCCTTTTTTTGAGCT<br>GTCTGCATGTGTGTCAGC |
| 4plexFuzzy6.2: linker3-AtU6.26-gRNA3-scaffold-linker4-AtU3b-gRNA4-scaffoldtail | GAGCTGTCTGCATGTGTGTCAGCGGAGTGATCAAAAGTCCCACATCGATCAGGTGATATAT<br>AGCAGCTTAGTTTATATAATGATAGAGTCGACATAGCGATTGTGGCTCAATGGGAGAGTGC<br>TGTTTCAGAGCTATGCTGGAAACAGCATAGCAAGTTGAAATAAGGCTAGTCCGTTATCAAC<br>TTGAAAAAGTGGCACCGAGTCGGTGCTTTTTTTGGACTGCGATTATGGAGCGTGCATTTCT<br>CCCACATCGCTCAAATCTAAACAAATCTTGTTGTATATATAACACTGAGGGAGCACCATTG<br>GTCAGCTTATATCATGAATTGCCAGTTTTAGAGCTAGAAATAGCAAG |

## Supplementary Text S1. Materials and Methods

### Binary vector construction

Pol III promoter sequences were retrieved from published studies (Li et al., 2013; Jacobs et al., 2015; Ma et al., 2015; Ren et al., 2016; Bernard et al., 2019; Charrier et al., 2019; Dai et al., 2021) or from the *Populus trichocarpa* v3.1 genome (Tuskan et al., 2006) available at Phytozome v13 (Goodstein et al., 2012) and provided in Supplementary Table S1. We first modified the pORE303N (Addgene ID 194438) (Ortega et al., 2023) by replacing the double CaMV35S promoter upstream of the human codon-optimized *Streptococcus pyogenes SpyCas9* with the *Arabidopsis thaliana* AtRps5A promoter from pKIR1.1 (Addgene ID 85758) (Tsutsui and Higashiyama, 2017). This was to avoid potential transcriptional silencing of the *mEGFP* (monomeric enhanced green fluorescent protein) reporter and *SpyCas9* if both were under control of the double CaMV35S promoter. A pair of *mEGFP*-targeting gRNA oligos with vector homology tails was Gibson-assembled into *Kpn*I-digested vector behind the *MtU6.6* promoter (352 bp) to produce p304N. To facilitate cloning of various Pol III promoters, we next generated a promoterless construct by digesting p304N with *Avr*II and *Spe*I for Gibson-assembly with an *mEGFP* gRNA-scaffold PCR fragment and a bridge oligo containing a built-in *Kpn*I site to produce p305N. The truncated *MtU6.6* (189 bp and 106 bp) promoter fragments were PCR amplified and cloned into *Kpn*I-digested and dephosphorylated p305N. Short 70 bp promoters were oligo-synthesized with vector overhangs, and Gibson-assembled either individually or as pools of 3-5 oligos into p305N at the *Kpn*I site.

A multiplex construct was designed to target the eight trichome-regulating *MYB* alleles in hybrid poplar (*Populus tremula* × *alba* IRNA 717-1B4)(Bewg et al., 2022) using four gRNAs driven by distinct U6 and U3 promoters. Inactivation of these *MYBs* requires expression of all four gRNAs and results in glabrous plants. To enable a direct comparison of promoter activities in multiplex editing, we selected four promoters of varying lengths to direct  gRNA expression. gRNA1, driven by the 352 bp *MtU6.6* promoter, targets *MYB138t1* (PtXaTreH.08G070600), *MYB186t2* (PtXaTreH.08G070900), and *MYB138t2* (PtXaTreH.08G071200); gRNA2, driven by the 101 bp *AtU3d* promoter, targets *MYB186t1* (PtXaTreH.08G070200) and *MYB186a* (PtXaAlbH.08G073700); gRNA3, driven by the 79 bp *AtU6.26* promoter, targets *MYB38t* (PtXaTreH.10G136900) and *MYB38a* (PtXaAlbH.10G130200); and gRNA4, driven by the 70 bp *AtU3d* promoter, targets MYB138a (PtXaAlbH.08G074000). The insert was synthesized as two overlapping DNA fragments (Twist Bioscience) (Supplementary Table S3) for direct assembly into the *Kpn*I-digested pORE303N vector (Ortega et al., 2023).

All Gibson assembly reactions were performed using the NEBuilder HiFi DNA Assembly Cloning Kit (New England Biolabs), with primers listed in Supplementary Table S3. All vectors were sequence-verified prior to transformation into *Agrobacterium tumefaciens* strain C58/pMP90 (Koncz and Schell, 1986). Multiplex cloning using oligo pools sometimes yielded erroneous clones containing unexpected insertions and/or deletions. Several of these clones were also tested to assess the mutational effects on promoter activity. All constructs will be made available through Addgene.

**Plant transformation**

*Nicotiana benthamiana* transformation was performed according to Horsch et al. (1989) with several modifications. Young (third and fourth) leaves from two-week-old seedlings were used as explants and MS salts and vitamins (instead of B5 vitamins) were used as the basal medium, with 200 mg/L Timentin to suppress *Agrobacterium* growth and 100 mg/L kanamycin for selection. Poplar (*Populus tremula* × *alba* IRNA 717-1B4) transformation and regeneration was performed as described (Ortega et al., 2023), with 3-day *Agrobacterium* cocultivation at 19°C. All experiments were performed using *mEGFP*-expressing reporter lines of *N. benthamiana* and poplar previously transformed with construct pH_35S/mEGFP (Addgene 135321) (Behrendorff et al., 2020) under selection of 10 mg/L hygromycin. All cultures were maintained at 22°C under a 16-h light/8-h dark photoperiod with Grolite FPV24 LED (Barron Lighting Group) at ~150 µmol $m^{-2}$ $s^{-1}$.

**Amplicon sequencing determination of mutations**

Young leaves from transgenic *N. benthamiana* and poplar plants in tissue culture (approximately 1.3-2 cm in length) were sampled for DNA card preparation according to Jia et al. (2021). The first PCR was performed using a DNA card punch (1 mm for poplar or 1.5 mm for *N. benthamiana*) as template, *mEGFP*-specific primers tailed with Illumina adapter sequences, and AccuStartII PCR ToughMix (Quantabio). Following gel electrophoresis, samples were diluted 10-50X and 1.5 µL was used as template with Illumina indexing primers and GoTaq Green Master Mix (Promega) in the second PCR. After gel electrophoresis, samples were pooled, purified using magnetic beads (Sergi Lab Supplies), and quality checked by gel electrophoresis and Qubit fluorometric quantification (Thermo Fisher Scientific). Amplicon libraries were sequenced on an Illumina MiSeq using Nano V2 flow cells with 500 cycles (PE250) at Georgia Genomics and Bioinformatics Core of the University of Georgia (RRID:SCR_010994). After demultiplexing, data were analyzed by the open source program AGEseq (Xue and Tsai, 2015) with 1% mismatch allowance for

*mEGFP* or 0.005% for the highly homologous trichome-*MYB* alleles. Mutation patterns were summarized in Supplementary Table S2.

### Microscopic analysis

*N. benthamiana* root tissues were examined for mEGFP fluorescence using an Olympus MVX10 fluorescence stereomicroscope equipped with a DP72 camera and cellSens Standard software (Olympus, v1.13) for photo documentation. Imaging of *N. benthamiana* leaf trichomes was conducted at the Biomedical Microscopy Core, University of Georgia, using a Zeiss LSM 880 upright confocal microscope equipped with ZEN Black software. GFP signal was detected using an argon excitation laser (488 nm) and an emission filter at 490–540 nm. Poplar leaf trichomes were visualized under a Leica M165FC dissecting microscope and photographed with a Leica DMC6200 digital camera.

### References

**Koncz C, Schell J** (1986) The promoter of TL-DNA gene 5 controls the tissue-specific expression of chimaeric genes carried by a novel type of *Agrobacterium* binary vector. Molecular and General Genetics **204:** 383-396

**Horsch RB, Fry J, Hoffmann N, Neidermeyer J, Rogers SG, Fraley RT** (1989) Leaf disc transformation. *In* SB Gelvin, RA Schilperoort, DPS Verma, eds, Plant Molecular Biology Manual. Springer Netherlands, Dordrecht, pp 63-71

**Tuskan GA, DiFazio S, Jansson S, Bohlmann J, Grigoriev I, Hellsten U, Putnam N, Ralph S, Rombauts S, Salamov A, Schein J, Sterck L, Aerts A, Bhalerao RR, Bhalerao RP, Blaudez D, Boerjan W, Brun A, Brunner A, Busov V, Campbell M, Carlson J, Chalot M, Chapman J, Chen GL, Cooper D, Coutinho PM, Couturier J, Covert S, Cronk Q, Cunningham R, Davis J, Degroeve S, Dejardin A, dePamphilis C, Detter J, Dirks B, Dubchak I, Duplessis S, Ehlting J, Ellis B, Gendler K, Goodstein D, Gribskov M, Grimwood J, Groover A, Gunter L, Hamberger B, Heinze B, Helariutta Y, Henrissat B, Holligan D, Holt R, Huang W, Islam-Faridi N, Jones S, Jones-Rhoades M, Jorgensen R, Joshi C, Kangasjarvi J, Karlsson J, Kelleher C, Kirkpatrick R, Kirst M, Kohler A, Kalluri U, Larimer F, Leebens-Mack J, Leple JC, Locascio P, Lou Y, Lucas S, Martin F, Montanini B, Napoli C, Nelson DR, Nelson C, Nieminen K, Nilsson O, Pereda V, Peter G, Philippe R, Pilate G, Poliakov A, Razumovskaya J, Richardson P, Rinaldi C, Ritland K, Rouze P, Ryaboy D, Schmutz J, Schrader J, Segerman B, Shin H, Siddiqui A, Sterky F, Terry A, Tsai CJ, Uberbacher E, Unneberg P, Vahala J, Wall K, Wessler S, Yang G, Yin T, Douglas C, Marra M, Sandberg G, Van de Peer Y, Rokhsar D** (2006) The genome of black cottonwood, Populus trichocarpa (Torr. & Gray). Science **313:** 1596-1604

**Goodstein DM, Shu S, Howson R, Neupane R, Hayes RD, Fazo J, Mitros T, Dirks W, Hellsten U, Putnam N, Rokhsar DS** (2012) Phytozome: a comparative platform for green plant genomics. Nucleic Acids Research **40:** D1178-D1186

**Li J-F, Norville JE, Aach J, McCormack M, Zhang D, Bush J, Church GM, Sheen J** (2013) Multiplex and homologous recombination–mediated genome editing in *Arabidopsis* and *Nicotiana benthamiana* using guide RNA and Cas9. Nature Biotechnology **31:** 688-691

**Jacobs TB, LaFayette PR, Schmitz RJ, Parrott WA** (2015) Targeted genome modifications in soybean with CRISPR/Cas9. BMC Biotechnology **15:** 16

**Ma X, Zhang Q, Zhu Q, Liu W, Chen Y, Qiu R, Wang B, Yang Z, Li H, Lin Y, Xie Y, Shen R, Chen S, Wang Z, Chen Y, Guo J, Chen L, Zhao X, Dong Z, Liu Y-G** (2015) A robust CRISPR/Cas9 system for convenient, high-efficiency multiplex genome editing in monocot and dicot plants. Molecular Plant **8:** 1274-1284

**Xue L-J, Tsai C-J** (2015) AGEseq: Analysis of genome editing by sequencing. Molecular Plant **8:** 1428-1430

**Ren C, Liu X, Zhang Z, Wang Y, Duan W, Li S, Liang Z** (2016) CRISPR/Cas9-mediated efficient targeted mutagenesis in Chardonnay (Vitis vinifera L.). Scientific reports **6:** 32289-32289

**Tsutsui H, Higashiyama T** (2017) pKAMA-ITACHI vectors for highly efficient CRISPR/Cas9-mediated gene knockout in *Arabidopsis thaliana*. Plant and Cell Physiology **58:** 46-56

**Bernard G, Gagneul D, Alves Dos Santos H, Etienne A, Hilbert J-L, Rambaud C** (2019) Efficient genome editing using CRISPR/Cas9 technology in chicory. International Journal of Molecular Sciences **20:** 1155

**Charrier A, Vergne E, Dousset N, Richer A, Petiteau A, Chevreau E** (2019) Efficient targeted mutagenesis in apple and first time edition of pear using the CRISPR-Cas9 system. Frontiers in Plant Science **10:** 40

**Behrendorff J, Borràs-Gas G, Pribil M** (2020) Antimicrobial solid media for screening non-sterile *Arabidopsis thaliana* seeds. Physiol Plant **169:** 586-599

**Dai X, Yang X, Wang C, Fan Y, Xin S, Hua Y, Wang K, Huang H** (2021) CRISPR/Cas9-mediated genome editing in Hevea brasiliensis. Industrial Crops and Products **164:** 113418

**Jia Z, Ding M, Nakano M, Hong K, Huang R, Becker D, Glazebrook J, Katagiri F, Han X, Tsuda K** (2021) DNA purification-free PCR from plant tissues. Plant Cell Physiol **62:** 1503-1505

**Bewg WP, Harding SA, Engle NL, Vaidya BN, Zhou R, Reeves J, Horn TW, Joshee N, Jenkins JW, Shu S, Barry KW, Yoshinaga Y, Grimwood J, Schmitz RJ, Schmutz J, Tschaplinski TJ, Tsai C-J** (2022) Multiplex knockout of trichome-regulating MYB duplicates in hybrid poplar using a single gRNA. Plant Physiology **189:** 516-526

**Ortega MA, Zhou R, Chen MSS, Bewg WP, Simon B, Tsai C-J** (2023) In vitro floral development in poplar: insights into seed trichome regulation and trimonoecy. New Phytologist **237:** 1078-1081
